# Burden and Outcomes of Cardiovascular Diseases in the ICU of a Nigerian Tertiary Hospital

**DOI:** 10.7759/cureus.90886

**Published:** 2025-08-24

**Authors:** Boma Oyan, Marcel F Jumbo, Sarah Abere

**Affiliations:** 1 Internal Medicine, Rivers State University Teaching Hospital, Port-Harcourt, NGA

**Keywords:** cardiovascular, critical care, icu, intensive cardiac care unit, mortality, nigeria

## Abstract

Introduction: The rising prevalence of cardiovascular diseases in Nigeria makes it imperative to develop specialised cardiac intensive care units (CICU) with uniquely trained staff for the management of critically ill cardiac cases that are currently being managed in the general ICU. This study aimed to describe the clinical characteristics and outcomes of patients with cardiac disease admitted into the ICU of the University of Port Harcourt Teaching Hospital.

Methods: This was a retrospective cross-sectional hospital-based study. The records of medical patients admitted into the ICU from January 2019 to December 2023 were studied. Patients’ biodata, clinical information, and outcomes were extracted. Data was analysed using SPSS version 25 (IBM Corp., Armonk, NY).

Results: Cardiac diseases accounted for 91 (28.0%) of the 325 medical patients admitted into the ICU with a mean age of 55.9±17.4years, and 46 (50.5%) were men. Hypertensive emergencies were the commonest cardiac indication for admission in 29 (31.9%) persons, followed by acute decompensated heart failure (ADHF) and pulmonary embolism in 24(26.4%) and 22 (24.2%) persons, respectively. Others included cardiogenic shock in nine (9.9%), unstable arrhythmias in five (5.5%), and acute myocardial infarction in two (2.2%) patients. A total of 50 (54.9%) persons admitted for cardiovascular disease died, with a significant relationship between increasing age (p=0.046), use of ionotropic/vasoactive medication (p=0.002), and use of mechanical ventilation (p=0.048) with mortality.

Conclusion: Hypertensive emergencies and ADHF emerged as significant contributors to cardiac morbidity and mortality in the ICU. This data may be useful to guide cardiac critical care redesign to improve patient outcomes.

## Introduction

Cardiovascular diseases (CVDs) remain the leading cause of death globally, responsible for an estimated 17.9 million deaths annually, representing 32% of all global deaths [1]. Low- and middle-income countries (LMICs), including Nigeria, bear a disproportionate share of this burden, accounting for over 75% of CVD-related deaths [2]. In Nigeria, the rising prevalence of hypertension, diabetes, and lifestyle-related risk factors has led to a growing incidence of acute cardiovascular emergencies, many of which require intensive care interventions [3].

Despite this growing burden, critical care infrastructure across much of sub-Saharan Africa, including Nigeria, remains underdeveloped. In particular, the availability of specialised cardiac ICUs (CICUs) is minimal; also, there is a chronic shortage of trained personnel and inadequate diagnostic and monitoring tools [4]. As a result, patients presenting with life-threatening cardiovascular conditions are typically managed within general ICUs, which may lack the focused expertise, equipment, and protocols needed to optimise outcomes for this complex patient population [5].

Critically ill cardiac patients admitted to the ICU often require advanced monitoring, mechanical ventilation, and hemodynamic support. However, the availability of such interventions and their outcomes in resource-limited settings have not been sufficiently studied. Furthermore, data on the specific clinical profiles, management strategies, and outcomes of cardiovascular ICU admissions in Nigerian tertiary hospitals are scarce. This knowledge gap makes it difficult to identify areas for improvement, allocate resources appropriately, or advocate effectively for specialised services and training [4,5].

This study aims to address this gap by providing a detailed review of cardiovascular disease-related ICU admissions over a five-year period at the University of Port Harcourt Teaching Hospital, a major tertiary referral centre in southern Nigeria. Specifically, it evaluates the burden, clinical characteristics, management approaches, and outcomes, particularly mortality rates among cardiovascular patients admitted to the general medical ICU.

By highlighting the scale and severity of cardiovascular ICU admissions in this setting, the study seeks to inform clinical practice, guide policy on ICU resource allocation, and advocate for the development of specialised cardiac critical care services in Nigeria and similar LMIC contexts.

## Materials and methods

This retrospective, cross-sectional, hospital-based study was conducted in the ICU of the University of Port Harcourt Teaching Hospital, a tertiary referral centre located in Port Harcourt, the capital of Rivers State in South-South Nigeria. The hospital serves an estimated population of 7.4 million residents within Rivers State, as well as patients from neighbouring states in the Niger Delta region. The study spanned five years, from January 2019 to December 2023, and included all adult patients aged 18 years and above who were admitted to the ICU with a medical diagnosis during this period. From this population, all patients with cardiovascular conditions were identified and included in the analysis. Patient selection was based on documentation in the ICU admission, discharge, and mortality registers. Detailed clinical data were extracted from case notes, nursing observation charts, and treatment records. These included demographic information, cardiovascular diagnoses, presenting symptoms, vital signs at admission, comorbidities, complications during ICU stay, therapeutic interventions, length of stay, and discharge outcomes. Diagnoses were recorded as noted by the supervising consultants and reflected the clinical assessments and decision-making documented in real-time during the ICU admission. Hypertensive emergencies refer to cases where patients present with markedly elevated blood pressures accompanied by clinical evidence of acute target organ damage. Conditions included under this category were hypertensive encephalopathy, acute aortic dissection, hypertensive crisis with acute kidney injury, and intracerebral haemorrhage, as identified by the supervising consultants. Cases of acute myocardial infarction (AMI) and acute decompensated heart failure (ADHF) were not grouped under hypertensive emergencies, as they were diagnosed and analysed separately when documented as primary clinical conditions. No formal sample size calculation was performed. Instead, all eligible cases within the five years were included, enhancing the robustness and representativeness of the findings within the limitations of available data.

Measurements

Clinical measurements were derived directly from routine documentation at the point of ICU admission. Random blood glucose levels were recorded using bedside glucometers, and hyperglycaemia was defined as values equal to or greater than 11.1 mmol/L, in line with World Health Organisation guidelines [6]. Blood pressure and oxygen saturation were measured using automated non-invasive monitors and pulse oximetry, respectively. Hypoxia was defined as peripheral oxygen saturation below 94% a commonly accepted clinical threshold [7].

Mean arterial pressure (MAP) was either recorded directly or calculated retrospectively using the standard formula [8], where SBP is systolic blood pressure and DBP is diastolic blood pressure:

$MAP = DBP + 1/3 * (SBP - DBP)$

Neurological status was assessed using the Glasgow Coma Scale (GCS), with coma defined as a GCS score of 5 or below [9]. Anaemia was identified using haemoglobin thresholds of <13 g/dL in men and <12 g/dL in women as defined by the WHO [10]. The presence of respiratory failure, chest infection, acute kidney injury, electrolyte abnormalities, and other complications was recorded based on clinical documentation and supporting investigations during the ICU stay. Therapeutic interventions such as the use of intravenous inotropic or vasopressor agents and mechanical ventilation were confirmed through treatment charts and nursing records.

Data were entered and analysed using the Statistical Package for the Social Sciences (SPSS) version 25 (IBM Corp, Armonk, NY). Descriptive statistics were used to summarise the data: categorical variables were presented as frequencies and percentages, while continuous variables were reported as means with standard deviations. Associations between categorical variables such as diagnosis, interventions, and outcome were tested using the chi-square test. Fisher’s exact test was applied when expected cell counts were fewer than five, following statistical best practices for small sample sizes [11]. Continuous variables such as mean arterial pressure were categorised into clinically relevant ranges to allow for categorical analysis. A p-value of less than 0.05 was considered statistically significant throughout the analysis [12].

## Results

During these five years, a total of 325 critically ill medical patients were admitted into the ICU of the University of Port Harcourt Teaching Hospital. The mean age was 53.7±17.9 years (range, 18 to 94 years), with a male-to-female ratio of 1.3, as 184 (56.6%) persons were men and 141 (43.4%) were women.

Out of this total number, 91 patients were admitted with critical cardiovascular diseases, which represented 28.0% of recorded cases. The mean age of the patients with cardiovascular disease was 55.9±17.4years (range of 18 to 94years), with a slight male preponderance, as 46 (50.5%) patients were men, as shown in Table 1.

**Table 1:** Age group and sex distribution of patients with cardiovascular disease in the ICU This table presents the age distribution of male-to-female patients admitted to the ICU with cardiovascular disease. Patients were grouped into three age categories: <45 years, 45 to 46 years, and ≥65 years. The distribution highlights a slight male predominance overall, with variations across age groups. Statistical comparison between sexes across age groups was assessed using the chi-square test. χ2= 3.688, p= 0.158

Age group (in years)	Sex		Total
	Male; n (%)	Female; n (%)	
<45	11(23.9)	15(33.3)	26
45 to 64	20(43.5)	11(24.4)	31
≥65	15(32.6)	19(42.2)	34
Total	46(100.0)	45(100.0)	91

Hypertensive emergencies were the commonest cardiovascular indication for admission into the ICU, as it was documented in 29 (31.9%) persons, followed by ADHF and pulmonary embolism (PE), which occurred in 24 (26.4%) and 22 (24.2%) persons, respectively. Other cardiac indications for admission included cardiogenic shock, which was reported in nine (9.9%) persons, unstable arrhythmias in five (5.5%) persons, and AMI in two (2.2%) patients.

As demonstrated in Table 2, ADHF occurred equally between men and women, while hypertensive emergencies, AMI, and cardiogenic shock were more common in men; PE and unstable arrhythmias occurred more in women.

**Table 2:** Sex distribution of the cardiovascular cases admitted into the ICU This table shows the distribution of specific cardiovascular diagnoses among male and female patients admitted to the ICU. Diagnoses include hypertensive emergencies, AMI, ADHF, cardiogenic shock, PE, and unstable arrhythmias. The relationship between diagnosis and sex was evaluated using Fisher's exact test. Fishers exact p=0.010 AMI: acute myocardial infarction; ADHF: acute decompensated heart failure; PE: pulmonary embolism

Cardiovascular diagnosis	Sex		Total
	Male; n(%)	Female; n(%)	
Hypertensive emergency	17(58.6)	12(41.4)	29
AMI	2(100.0)	0(0)	2
ADHF	12(50.0)	12(50.0)	24
Cardiogenic shock	8(88.9)	1(11.1)	9
PE	5(22.7)	17(77.3)	22
Unstable arrhythmias	2(40.0)	3(60.0)	5

AMI occurred solely in middle-aged persons, whereas cardiogenic shock occurred in middle-aged and elderly persons. A higher number of patients under the age of 45 presented with ADHF, hypertensive emergencies, and PE, as shown in Table 3.

**Table 3:** Age group distribution of the cardiovascular cases admitted into the ICU This table shows the distribution of cardiovascular conditions across three age groups: <45 years, 45-64 years, and ≥65 years. Each diagnosis category is presented as a frequency and percentage within each age group. The analysis illustrates how conditions such as ADHF and PE occurred across different age strata. Fisher's exact test was used to assess statistical association. AMI: acute myocardial infarction; ADHF: acute decompensated heart failure; PE: pulmonary embolism Fisher's exact (p=0.32).

Cardiovascular diagnosis	Age group (in years)			Total
	<45; n(%)	45-64; n (%)	≥65; n(%)	
Hypertensive emergencies	6(20.7)	14(48.3)	9(31.0)	29
AMI	0(0)	2(100.0)	0(0)	2
ADHF	11(45.8)	3(12.5)	10(41.7)	24
Cardiogenic shock	0(0.0)	6(66.7)	3(33.3)	9
PE	7(31.8)	5(22.7)	10(45.5)	22
Unstable arrhythmias	2(40.0)	1(20.0)	2(40.0)	5

Almost half of the patients had a history of pre-existing cardiac disease, and hypertension was the most common comorbidity seen in 61.5%. On admission into the ICU, the more frequent clinical features documented included hyperglycaemia in 60.4%, hypoxia in 52.7% while fever and elevated systemic mean arterial pressures were found in 40.7% of the patients. After admission to the ICU, a total of 71 (78.0%) patients were treated with intravenous vasoactive and/or ionotropic medications, and 44 (48.4%) with mechanical ventilation. Complications that occurred during admission included anaemia in 44.0%, chest infection in 29.7% and respiratory failure in 28.6% patients. These clinical characteristics are shown in Table 4 below.

**Table 4:** Characteristics and complications of patients with cardiovascular diseases in the ICU This table summarises the clinical presentation, underlying comorbidities, and complications that occurred during ICU admission for patients with cardiovascular disease. Parameters include presenting features (e.g., hyperglycaemia, hypoxia), physiological measures (e.g., MAP, pulse rate), and documented complications such as respiratory failure and sepsis. Data are shown as absolute frequencies and percentages. Mean values are reported where applicable. * mean ± SD RBG: random blood glucose; MAP: mean arterial pressure; SBP: systolic blood pressure; DBP: diastolic blood pressure; SPO2: oxygen saturation; GCS: Glasgow Coma Scale

Parameters	n (%)
Comorbidities	
Pre-existing cardiac disease	39(42.9)
Hypertension	56(61.5)
Diabetes mellitus	24(26.4)
Clinical features at presentation	
Fever	37(40.7)
Hyperglycaemia (RBG ≥11.1mmol/l)	55(60.4)
Mean RBG	12.2±4.5*
Hypoxia (SPO2<95%)	48(52.7)
Coma (GCS≤5)	21(23.1)
MAP<60mmHg	15(16.5)
MAP>100mmHg	37(40.7)
Mean SBP	130±25.9*
Mean DBP	78±17.5*
MAP	94±21.2*
Pulse rate	89.2±21.7*
Complications during admission	
Chest infection	27(29.7)
Sepsis	19(20.9)
Pressure ulcers	4(4.4)
Respiratory failure	26(28.6)
Electrolyte derangements	23(25.3)
Anaemia	40(44.0)
Acute kidney injury	12(13.2)

**Figure 1:**
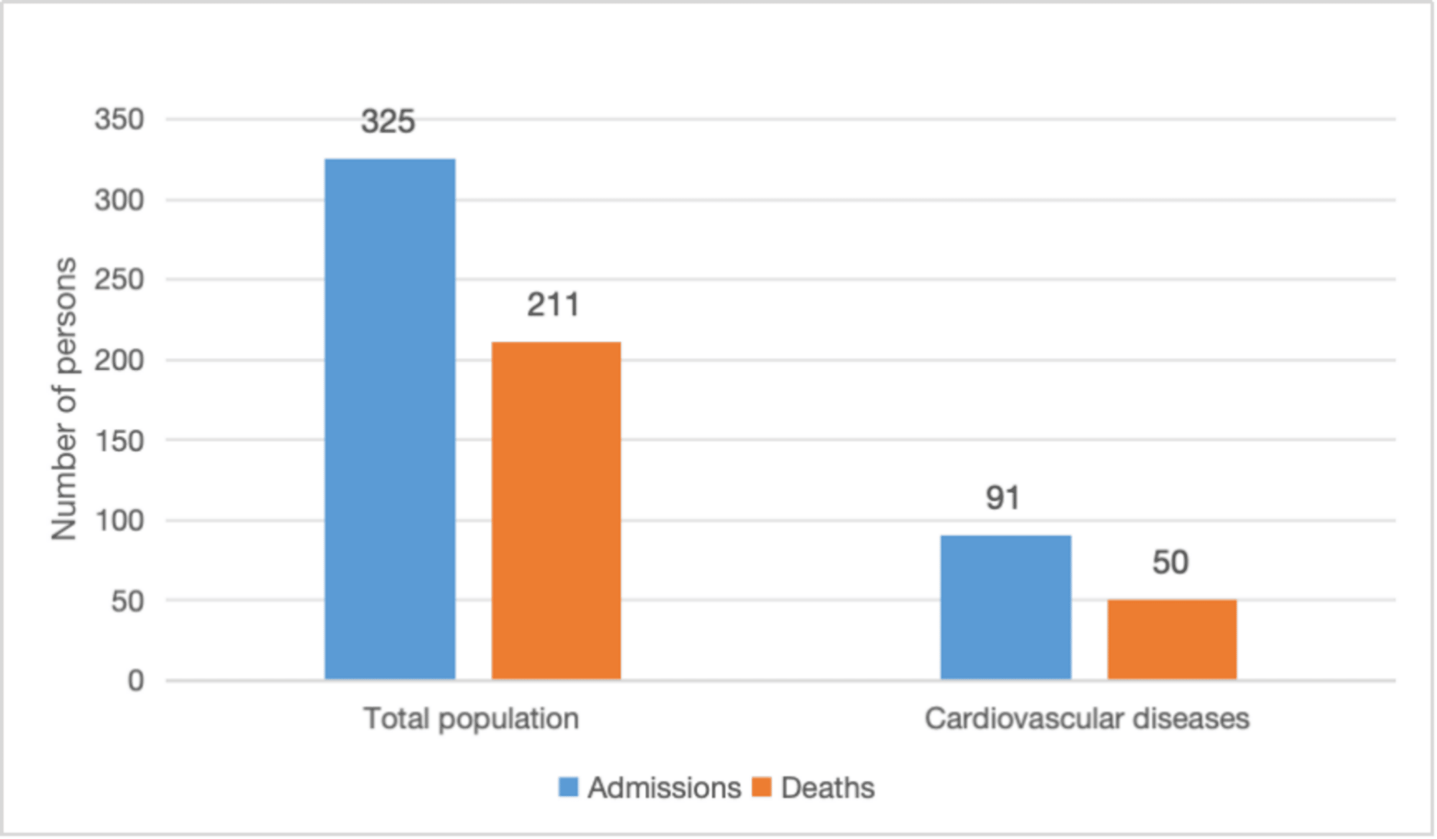
Total and cardiovascular diseases admissions and deaths in the ICU This figure illustrates the total number of medical ICU admissions over the study period, highlighting the proportion of cardiovascular admissions and the  corresponding number if deaths. It provides a visual summary of the burden of cardiovascular disease relative to overall ICU activity and mortality.

Of the total number of medical cases admitted into the ICU, 211 (64.9%) died, of which 50 persons admitted for cardiac disease died, which represents 23.7% of all medical deaths in the ICU and 54.9% of total cardiac disease admissions (Figure 1). The remaining 41 (45.1%) persons with cardiac diseases were discharged to the general medical wards before their discharge from the hospital, and the duration of stay in the ICU before discharge or death ranged from one day to 19 days, with a median of three days (IQR=1 to 6 days).

The clinical characteristics and management outcomes for patients with CVDs who ultimately died in the ICU are summarised in Table 5. Across the full age spectrum, age category was significantly associated with mortality (chi-square test), with younger patients having better outcomes compared to older patients. There was no significant difference in mortality between men and women. The majority of deaths occurred in patients with hypertensive emergencies and PE. Those admitted for AMI all survived; however, almost all admitted for unstable arrhythmias died. Nevertheless, these differences were not statistically significant. The requirement for intravenous ionotropic/vasoactive medication as well as mechanical ventilation was significantly associated with mortality, as about two-thirds of the patients who required these interventions died during admission.

**Table 5:** Patients’ characteristics associated with death in the ICU This table compares the demographic, clinical, and management characteristics of cardiovascular patients who died versus those who survived their ICU stay. Variables include age group, sex, diagnosis, MAP, comorbidities, and use of critical care interventions. Chi-square tests were used for categorical comparisons where cell counts were adequate, including analysis across the full age spectrum (which showed a significant association between older age and mortality). Fisher’s exact test was applied for cardiovascular diagnosis due to small subgroup sizes (marked with *). Statistically significant associations are indicated by p-values <0.05. CV: cardiovascular; AMI: acute myocardial infarction; ADHF: acute decompensated heart failure; PE: pulmonary embolism, IV: intravenous

Parameter	Dead; n (%)	Discharged; n (%)	Total	χ2	p-value
Age group (in years)				6.153	0.046
<45	9(34.6)	17(65.4)	26		
45-64	19(61.3)	12(38.7)	31		
≥65	22(64.7)	12(35.3)	34		
Sex				0.093	0.834
Male	26(56.5)	20(43.5)	46		
Female	24(53.3)	21(46.7)	45		
Pre-existing heart disease				0.033	0.512
Yes	21(53.8)	18(41.2)	39		
No	29(55.8)	23(44.2)	52		
CV indication for admission					0.535*
Hypertensive emergency	16(55.2)	13(44.8)	29		
AMI	0(0)	2(100.0)	2		
ADHF	12(50.0)	12(50.0)	24		
Cardiogenic shock	5(55.6)	4(44.4)	9		
PE	13(59.1)	9(40.9)	22		
Unstable arrhythmias	4(80.0)	1(20.0)	5		
IV ionotropic / vasoactive medication				9.285	0.002
Yes	45(63.4)	26(36.7)	71		
No	5(25.0)	15(75.0)	20		
Mechanical ventilation				6.04	0.048
Yes	28(63.6)	16(36.4)	44		
No	22(46.8)	25(53.2)	47		
Mean arterial pressure				2.299	0.317
<60mmHg	10(66.7)	5(33.3)	15		
60-100mmHg	23(59.0)	16(41.0)	39		
>100mmHg	17(45.9)	20(54.1)	37		
Acute kidney injury				1.137	0.763
Present	6(50.0)	6(50.0)	12		
Absent	44(55.7)	35(44.3)	79		

## Discussion

This study provides compelling evidence of the significant burden and poor outcomes associated with CVDs in the ICU setting of a tertiary hospital in Nigeria. With CVDs accounting for 28.0% of all medical ICU admissions and over half of these patients not surviving their ICU stay, our findings underscore the critical and growing role of CVDs in ICU case mix and mortality in sub-Saharan Africa.

Consistent with existing literature, hypertensive emergencies were the most frequent cause of ICU admission among cardiovascular cases, followed by ADHF and PE [2,13,14]. The prominence of hypertensive crises reflects the widespread burden of poorly controlled hypertension in Nigeria, where population-level awareness, treatment, and control rates remain low [15]. ADHF and PE, both of which are late-stage or acute manifestations of underlying cardiovascular pathology, further reflect a trend of late hospital presentation and limited access to primary or specialist care [16].

The high ICU mortality rate observed among CVD patients is comparable to other reports from resource-limited settings, where overall critical care outcomes tend to be worse due to infrastructural and workforce constraints [17,18]. Notably, the need for mechanical ventilation and intravenous vasoactive/inotropic support was independently associated with mortality. These interventions, while lifesaving, likely indicate the severity of illness at presentation and may also reflect the lack of intermediary support or monitoring systems that would allow for earlier interventions [19].

Despite improvements in cardiovascular medicine globally, outcomes in LMICs such as Nigeria remain poor due to gaps in access to care, limited ICU capacity, and a shortage of trained intensivists and cardiologists [20]. Our findings support the growing call for dedicated CICUs or cardiac high-dependency units that are equipped with appropriate monitoring tools, staffed with personnel trained in advanced cardiovascular life support, and integrated into multidisciplinary care systems [21,22].

Younger patients (<45 years) demonstrated significantly better survival, suggesting a potential age-related resilience, but also raising questions about differential patterns of disease burden and access to care. Sex was not significantly associated with outcomes, indicating that male and female patients face similar risks once critically ill. Importantly, of the two patients who were admitted for AMI, both survived. While the small number precludes any firm conclusions, this observation may suggest potential benefits from early recognition and targeted management in selected cases [23].

Implications for clinical practice

This study has several practical implications. First, it highlights the urgent need for health system strengthening with a focus on cardiovascular disease prevention and emergency preparedness. The establishment of specialised CICUs within tertiary centres can facilitate protocol-driven management, optimise resource allocation, and improve survival outcomes. Second, continuous training of ICU staff in cardiovascular critical care and early recognition of decompensating patients is essential. Third, strengthening community-based screening and primary care management of hypertension and heart failure can reduce the frequency of preventable ICU admissions.

Limitations

This retrospective single-centre study has several limitations, including selection bias, which may affect its generalizability to other settings. The accuracy of the findings relies on the completeness of clinical documentation; missing data resulted in the exclusion of some cases. Standardised illness severity scores were not included, as they were not routinely documented and could not be accurately reconstructed retrospectively. Resource limitations, such as a shortage of trained personnel, inadequate diagnostic and monitoring tools, and insufficient critical care infrastructure, may have influenced management decisions and outcomes. Diagnoses such as cardiogenic shock and AMI were analysed as separate categories based on their documentation; however, the underlying causes were not consistently recorded, which limits deeper clinical interpretation. Additionally, the small number of cases in certain subgroups, such as AMI and arrhythmias, restricts the strength of the conclusions. The absence of a control group and a lack of long-term follow-up data after ICU discharge further constrain broader comparisons and insights into recovery.

## Conclusions

This five-year retrospective analysis provides rare, locally generated evidence on the burden, clinical characteristics, and outcomes of cardiovascular disease in a Nigerian ICU; a topic for which published data are extremely limited. Cardiovascular disease accounted for more than a quarter of all medical ICU admissions, with mortality exceeding half of those admitted, underscoring its major contribution to critical illness in this setting. The findings highlight urgent needs for improved early detection, timely intervention, and expansion of critical care capacity, particularly through the development of dedicated cardiac intensive care services. While resource limitations and retrospective design constrain the depth of analysis, this study offers a valuable benchmark for clinicians, policymakers, and researchers seeking to strengthen cardiovascular critical care in Nigeria and similar low-resource environments.
